
JOURNAL INFORMATION
==============================
NLM Title Abbreviation: ScientificWorldJournal
Journal ID: ScientificWorldJournal
NLM Journal ID: 101131163
Journal ID: TSWJ

The Scientific World Journal

ISSN: 2356-6140
EISSN: 1537-744X
Publisher: Wiley

ARTICLE INFORMATION
==============================
PMCID: PMC6083977
PMID: 30147579
DOI: 10.1100/tsw.2001.208
Article ID: 182904
Article version: 1
Subjects: Short Report

G1-CDK Activity Is Required for Both Proliferation and Viability of Myeloid/Erythroid Progenitors: A Critical Function of the v-Erba Oncoprotein

Hofmann Johannes *
Sykora Martina
Beug Hartmut
Research Institute of Molecular Pathology, A-1030 Vienna, Austria
*Johannes Hofmann: hofmann@nt.imp.univie.ac.at
Publication date: 2001
Electronic publication date: 2001 Dec 5
Volume: 1
First page: 99
Last page: 99
Copyright: Copyright © 2001 Johannes Hofmann et al.
Copyright year: 2001
License: This is an open access article distributed under the Creative Commons Attribution License, which permits unrestricted use, distribution, and reproduction in any medium, provided the original work is properly cited.
License URL: https://creativecommons.org/licenses/by/3.0/

==============================
Miami *Nature Biotechnology* Short Reports
*TheScientificWorld* (2001) 1 (S3), 99SR
ISSN 1532-2246; DOI 10.1100/tsw.2001.208

### G1-CDK ACTIVITY IS REQUIRED FOR BOTH PROLIFERATION AND VIABILITY OF MYELOID/ERYTHROID PROGENITORS: A CRITICAL FUNCTION OF THE V-ERBA ONCOPROTEIN

Johannes Hofmann*, Martina Sykora, and Hartmut Beug

Institute of Molecular Pathology, Dr. Bohr Gasse 7, 1030 Vienna, Austria
* hofmann@nt.imp.univie.ac.at

**INTRODUCTION**. Cytokine deprivation of hematopoietic progenitors is associated with rapid down-regulation of G1-Cdk activity, cell cycle arrest, and apoptosis. It is poorly understood how the apoptotic machinery of progenitor cells senses the absence of specific cytokines. Here we address an essential role of G1-Cdk activity in cytokine-mediated survival. Our data further suggest that the antiapoptotic activity of the v-ErbA oncogene in the absence of survival signals requires G1-Cdk activity.

**RESULTS**. We have previously shown that specific inhibition of G1-Cdk activity results in apoptotic cell death of both myeloid and erythroid progenitors in the presence of saturating cytokine levels. This cell death is indistinguishable from apoptosis caused by cytokine withdrawal by a number of criteria. In contrast, specific cell cycle arrest in G2/M by down-regulation of PLK (polo-like kinasis) activity does not affect viability. v-erbA infected primary erythroid progenitors (1,3) undergo significantly delayed apoptosis after survival factor withdrawal. Unlike the primary parental cells, v-erbA cells can be restimulated with growth factors (indicated by arrow in Fig. 1A) after extended starvation (Fig. 1A, black diamonds). Starved v-erbA cells retain cyclin E levels and Cdk2 associated histone H1 activity in the absence of cell proliferation, whereas primary cells lose both cyclin E protein and Cdk2 kinase activity (Fig. 1B). We demonstrate that the sustained Cdk2 kinase activity is essential for extended viability in starved v-erbA cells.

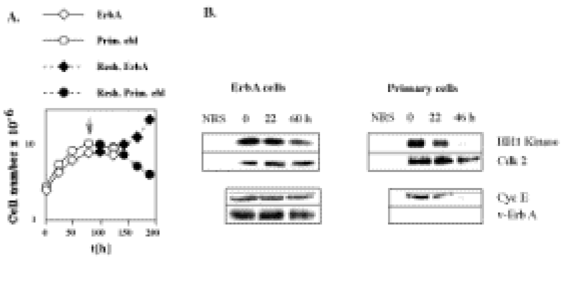

**Fig. 1.** v-erbA expression in growth factor deprived primary erythroblasts delays apoptosis and retains Cdk2 associated kinase activity.

**DISCUSSION**. Our data demonstrate a critical role of G1-Cdk activity for cytokine mediated survival as well as for v-erbA mediated delay of apoptosis in the absence of survival factors.

These observations may be relevant to cytostatic treatment regimens used in the clinic, which often severely affect hematopoietic maturation.

**REFERENCES.**

1. Beug, H., Bauer, A., Dolznig, H., von Lindern, M., Lobmayer, L., Mellitzer, G., Steinlein, P., Wessely, O., and Mullner, E. (1996) Biochim. Biophys. Acta 1288, M35-47
2. Gandrillon, O., Ferrand, N., Michaelle, J., Roze, L., Zile, M., and Samarut, J. (1994) Oncogene 9, 749-758
